# Data-driven discovery of coordinates and governing equations

**DOI:** 10.1073/pnas.1906995116

**Published:** 2019-10-21

**Authors:** Kathleen Champion, Bethany Lusch, J. Nathan Kutz, Steven L. Brunton

**Affiliations:** ^a^Department of Applied Mathematics, University of Washington, Seattle, WA 98195;; ^b^Leadership Computing Facility, Argonne National Laboratory, Lemont, IL 60439;; ^c^Department of Mechanical Engineering, University of Washington, Seattle, WA 98195

**Keywords:** model discovery, dynamical systems, machine learning, deep learning

## Abstract

The discovery of governing equations from scientific data has the potential to transform data-rich fields that lack well-characterized quantitative descriptions. Advances in sparse regression are currently enabling the tractable identification of both the structure and parameters of a nonlinear dynamical system from data. The resulting models have the fewest terms necessary to describe the dynamics, balancing model complexity with descriptive ability, and thus promoting interpretability and generalizability. This provides an algorithmic approach to Occam’s razor for model discovery. However, this approach fundamentally relies on an effective coordinate system in which the dynamics have a simple representation. In this work, we design a custom deep autoencoder network to discover a coordinate transformation into a reduced space where the dynamics may be sparsely represented. Thus, we simultaneously learn the governing equations and the associated coordinate system. We demonstrate this approach on several example high-dimensional systems with low-dimensional behavior. The resulting modeling framework combines the strengths of deep neural networks for flexible representation and sparse identification of nonlinear dynamics (SINDy) for parsimonious models. This method places the discovery of coordinates and models on an equal footing.

## Significance

Governing equations are essential to the study of physical systems, providing models that can generalize to predict previously unseen behaviors. There are many systems of interest across disciplines where large quantities of data have been collected, but the underlying governing equations remain unknown. This work introduces an approach to discover governing models from data. The proposed method addresses a key limitation of prior approaches by simultaneously discovering coordinates that admit a parsimonious dynamical model. Developing parsimonious and interpretable governing models has the potential to transform our understanding of complex systems, including in neuroscience, biology, and climate science.

Governing equations are of fundamental importance across all scientific disciplines. Accurate models allow for understanding of physical processes, which in turn gives rise to an infrastructure for the development of technology. The traditional derivation of governing equations is based on underlying first principles, such as conservation laws and symmetries, or from universal laws, such as gravitation. However, in many modern systems, governing equations are unknown or only partially known, and recourse to first-principles derivations is untenable. Instead, many of these systems have rich time-series data due to emerging sensor and measurement technologies (e.g., in biology and climate science). This has given rise to the new paradigm of data-driven model discovery, which is the focus of intense research efforts (1–14). A central tension in model discovery is the balance between model efficiency and descriptive capabilities. Parsimonious models strike this balance, having the fewest terms required to capture essential interactions (1, 3, 8, 10, 15), thus promoting interpretability and generalizability. Obtaining parsimonious models is fundamentally linked to the coordinate system in which the dynamics are measured. Without proper coordinates, standard approaches may fail to discover simple dynamical models. In this work, we simultaneously discover effective coordinates via a custom autoencoder (16–18), along with the parsimonious dynamical system model via sparse regression in a library of candidate terms (8). The joint discovery of models and coordinates is critical for understanding many modern systems.

Numerous recent approaches leverage neural networks to model time-series data (18–26). When interpretability and generalizability are primary concerns, it is important to identify parsimonious models that have the fewest terms required to describe the dynamics, which is the antithesis of neural networks whose parameterizations are exceedingly large. A breakthrough approach used symbolic regression to learn the form of dynamical systems and governing laws from data (1, 3). Sparse identification of nonlinear dynamics (SINDy) (8) is a related approach that uses sparse regression to find the fewest terms in a library of candidate functions required to model the dynamics. Because this approach is based on a sparsity-promoting linear regression, it is possible to incorporate partial knowledge of the physics, such as symmetries, constraints, and conservation laws (27). Successful modeling requires that the dynamics are measured in a coordinate system where they may be sparsely represented. While simple models may exist in one coordinate system, a different coordinate system may obscure these parsimonious representations. For modern applications of data-driven discovery, there is no reason to believe that we measure the correct variables to admit a simple representation of the dynamics. This motivates the present study to enable systematic and automated discovery of coordinate transformations that facilitate this sparse representation.

The challenge of discovering an effective coordinate system is as fundamental and important as model discovery. Many key scientific breakthroughs were enabled by the discovery of appropriate coordinate systems. Celestial mechanics, for instance, was revolutionized by the heliocentric coordinate system of Copernicus, Galileo, and Kepler, thus displacing Ptolemy’s doctrine of the perfect circle, which was dogma for more than a millennium. The Fourier transform was introduced to simplify the representation of the heat equation, resulting in a sparse, diagonal, decoupled linear system. Eigen-coordinates have been used more broadly to enable sparse dynamics, for example in quantum mechanics and electrodynamics, to characterize energy levels in atoms and propagating modes in waveguides, respectively. Principal component analysis (PCA) is one of the most prolific modern coordinate discovery methods, representing high-dimensional data in a low-dimensional linear subspace. Nonlinear extensions of PCA have been enabled by a neural network architecture, called an autoencoder (16, 17, 28). However, PCA and autoencoders generally do not take dynamics into account and, thus, may not provide the right basis for parsimonious dynamical models. In related work, Koopman analysis seeks coordinates that linearize nonlinear dynamics (29); while linear models are useful for prediction and control, they cannot capture the full behavior of many nonlinear systems. Thus, it is important to develop methods that combine simplifying coordinate transformations and nonlinear dynamics. We advocate for a balance between these approaches, identifying coordinate transformations where only a few nonlinear terms are present, as in near-identity transformations and normal forms.

In this work we present a method to discover nonlinear coordinate transformations that enable parsimonious dynamics. Our method combines a custom autoencoder network with a SINDy model for parsimonious nonlinear dynamics. The autoencoder enables the discovery of reduced coordinates from high-dimensional data, with a map back to reconstruct the full system. The reduced coordinates are found along with nonlinear governing equations for the dynamics in a joint optimization. We demonstrate the ability of our method to discover parsimonious dynamics on 3 examples: a high-dimensional spatial dataset with dynamics governed by the chaotic Lorenz system, the nonlinear pendulum, and a spiral wave resulting from the reaction–diffusion equation. These results demonstrate how to focus neural networks to discover interpretable dynamical models. Critically, the proposed method provides a mathematical framework that places the discovery of coordinates and models on equal footing.

## Background

We review the SINDy (8) algorithm, which is a regression technique for extracting parsimonious dynamics from time-series data. The method takes snapshot data $x\left(t\right)\in R^{n}$ and attempts to discover a best-fit dynamical system with as few terms as possible:

$$\frac{d}{dt}x\left(t\right)=f\left(x\left(t\right)\right).$$ [1]

The state of the system x evolves in time t, with dynamics constrained by the function f. We seek a parsimonious model for the dynamics, resulting in a function f that contains only a few active terms: It is sparse in a basis of possible functions. This is consistent with our extensive knowledge of a diverse set of evolution equations used throughout the physical, engineering, and biological sciences. Thus, the types of functions that compose f are typically known from modeling experience.

SINDy frames model discovery as a sparse regression problem. If snapshot derivatives are available, or can be calculated from data, the snapshots are stacked to form data matrices $X={\left[x_{1}\;x_{2}\;\cdots \;x_{m}\right]}^{T}$ and $\dot{X}={\left[{\dot{x}}_{1}\;{\dot{x}}_{2}\;\cdots \;{\dot{x}}_{m}\right]}^{T}$ with $X,\dot{X}\in R^{m\times n}$. Although f is unknown, we can construct an extensive library of p candidate functions $\Theta \left(X\right)=\left[\theta_{1}\left(X\right)\cdots \theta_{p}\left(X\right)\right]\in R^{m\times p}$, where each $\theta_{j}$ is a candidate model term. We assume m≫p so the number of data snapshots is larger than the number of library functions; it may be necessary to sample transients and multiple initial conditions to improve the condition number of Θ. The choice of basis functions typically reflects some knowledge about the system of interest: A common choice is polynomials in x as these are elements of many canonical models. The library is used to formulate an overdetermined linear system

$$\dot{X}=\Theta \left(X\right)\Xi ,$$

where the unknown matrix $\Xi =\left(\xi_{1}\;\xi_{2}\;\cdots \;\xi_{n}\right)\in R^{p\times n}$ is the set of coefficients that determine the active terms from Θ(X) in the dynamics f. Sparsity-promoting regression is used to solve for Ξ that result in parsimonious models, ensuring that Ξ, or more precisely each $\xi_{j}$, is sparse and only a few columns of Θ(X) are selected. For high-dimensional systems, the goal is to identify a low-dimensional state z=φ(x) with dynamics $\dot{z}=g\left(z\right)$, as in Eq. **2**. The standard SINDy approach uses a sequentially thresholded least-squares algorithm to find the coefficients (8), which is a proxy for $\ell_{0}$ optimization (30) and has convergence guarantees (31). Yao and Bollt (2) previously formulated system identification as a similar linear inverse problem without including sparsity, resulting in models that included all terms in Θ. In either case, an appealing aspect of this model discovery formulation is that it results in an overdetermined linear system for which many regularized solution techniques exist. Thus, it provides a computationally efficient counterpart to other model discovery frameworks (3).

SINDy has been widely applied to identify models for fluid flows (27), optical systems (32), chemical reaction dynamics (33), convection in a plasma (34), and structural modeling (35) and for model predictive control (36). There are also a number of theoretical extensions to the SINDy framework, including for identifying partial differential equations (10, 37), and models with rational function nonlinearities (38). It can also incorporate partially known physics and constraints (27). The algorithm can also be reformulated to include integral terms for noisy data (39) or handle incomplete or limited data (40, 41). The selected modes can also be evaluated using information criteria for model selection (42). These diverse mathematical developments provide a mature framework for broadening the applicability of the model discovery method.

### Neural Networks for Dynamical Systems.

The success of neural networks (NNs) on image classification and speech recognition has led to the use of NNs to perform a wide range of tasks in science and engineering (17). One recent focus has been the use of NNs to study dynamical systems, which has a surprisingly rich history (43). In addition to improving solution techniques for systems with known equations (24–26), deep learning has been used to understand and predict dynamics for complex systems with unknown equations (18–23). Several methods have trained NNs to predict dynamics, including a time-lagged autoencoder which takes the state at time t as input data and uses an autoencoder-like structure to predict the state at time t+τ (21). Other approaches use a recurrent architecture, particularly long short-term memory (LSTM) networks, for applications involving sequential data (44). LSTMs have recently been used for forecasting of chaotic dynamical systems (20). Reservoir computing has also enabled impressive predictions (13). Autoencoders are increasingly being leveraged for dynamical systems because of their close relationship to other dimensionality reduction techniques (28, 45–47).

Another class of NNs uses deep learning to discover coordinates for Koopman analysis. Koopman theory seeks to discover coordinates that linearize nonlinear dynamics (29). Methods such as dynamic mode decomposition (DMD) (4, 5, 9), extended DMD (48), and time-delay DMD (49) build linear models for dynamics, but these methods rely on a proper set of coordinates for linearization. Several recent works have focused on the use of deep-learning methods to discover the proper coordinates for DMD and extended DMD (22, 23). Other methods seek to learn Koopman eigenfunctions and the associated linear dynamics directly using autoencoders (18). While autoencoders are particularly useful when reconstruction of the original state space is necessary, there are many applications in which full reconstruction is unnecessary. Koopman analysis and its combination with neural networks have also shown impressive results for use in such forecasting applications (19, 50).

Despite their widespread use, NNs face 3 major challenges: generalization, extrapolation, and interpretation. The hallmark success stories of NNs (computer vision and speech, for instance) have been on datasets that are fundamentally interpolatory in nature. The ability to extrapolate, and as a consequence generalize, is known to be an underlying weakness of NNs. This is especially relevant for dynamical systems and forecasting, which is typically an extrapolatory problem by nature. Thus models trained on historical data will generally fail to predict future events that are not represented in the training set. An additional limitation of deep learning is the lack of interpretability of the resulting models. While attempts have been made to interpret NN weights, network architectures are typically complicated with the number of parameters (or weights) far exceeding the original dimension of the dynamical system. The lack of interpretability also makes it difficult to generalize models to new datasets and parameter regimes. However, NN methods still have the potential to learn general, interpretable dynamical models if properly constrained or regularized. In addition to methods for discovering linear embeddings (18), deep learning has also been used for parameter estimation of partial differential equations (PDEs) (24, 25).

## SINDy Autoencoders

**Fig. 1.**
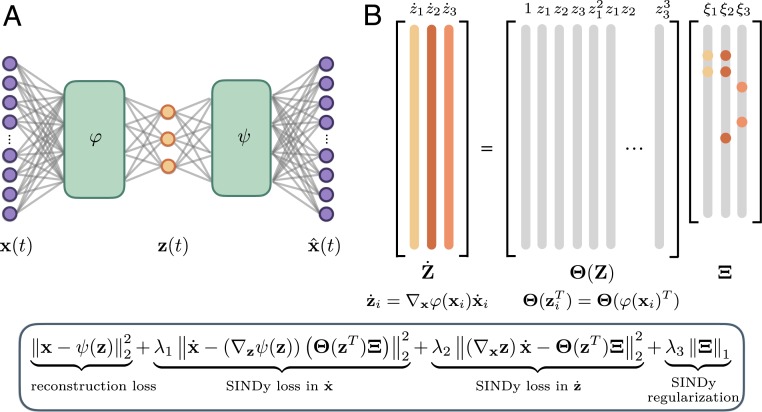
Schematic of the SINDy autoencoder method for simultaneous discovery of coordinates and parsimonious dynamics. (*A*) An autoencoder architecture is used to discover intrinsic coordinates z from high-dimensional input data x. The network consists of 2 components: an encoder φ(x), which maps the input data to the intrinsic coordinates z, and a decoder ψ(z), which reconstructs x from the intrinsic coordinates. (*B*) A SINDy model captures the dynamics of the intrinsic coordinates. The active terms in the dynamics are identified by the nonzero elements in Ξ, which are learned as part of the NN training. The time derivatives of z are calculated using the derivatives of x and the gradient of the encoder φ. *Inset* shows the pointwise loss function used to train the network. The loss function encourages the network to minimize both the autoencoder reconstruction error and the SINDy loss in z and x. $L_{1}$ regularization on Ξ is also included to encourage parsimonious dynamics.

We present a method for the simultaneous discovery of sparse dynamical models and coordinates that enable these simple representations. Our aim is to leverage the parsimony and interpretability of SINDy with the universal approximation capabilities of deep neural networks (51) to produce interpretable and generalizable models capable of extrapolation and forecasting. Our approach combines a SINDy model and a deep autoencoder network to perform a joint optimization that discovers intrinsic coordinates which have an associated parsimonious nonlinear dynamical model. The architecture is shown in Fig. 1. We again consider dynamical systems of the form **1**. While this dynamical model may be dense in terms of functions of the original measurement coordinates x, our method seeks a set of reduced coordinates $z\left(t\right)=\varphi \left(x\left(t\right)\right)\in R^{d}$ (d≪n) with an associated dynamical model

$$\frac{d}{dt}z\left(t\right)=g\left(z\left(t\right)\right)$$ [2]

that provides a parsimonious description of the dynamics; i.e., g contains only a few active terms. Along with the dynamical model, the method provides coordinate transforms φ,ψ that map the measurements to intrinsic coordinates via z=φ(x) (encoder) and back via x≈ψ(z) (decoder).

The coordinate transformation is achieved using an autoencoder network architecture. The autoencoder is a feedforward neural network with a hidden layer that represents the intrinsic coordinates. Rather than performing a task such as prediction or classification, the network is trained to output an approximate reconstruction of its input, and the restrictions placed on the network architecture (e.g., the type, number, and size of the hidden layers) determine the properties of the intrinsic coordinates (17); these networks are known to produce nonlinear generalizations of PCA (16). A common choice is that the dimensionality of the intrinsic coordinates z, determined by the number of units in the corresponding hidden layer, is much lower than that of the input data x: In this case, the autoencoder learns a nonlinear embedding into a reduced latent space. Our network takes measurement data $x\left(t\right)\in R^{n}$ from a dynamical system as input and learns intrinsic coordinates $z\left(t\right)\in R^{d}$, where d≪n is chosen as a hyperparameter prior to training the network.

While autoencoders can be trained in isolation to discover useful coordinate transformations and dimensionality reductions, there is no guarantee that the intrinsic coordinates learned will have associated sparse dynamical models. We require the network to learn coordinates associated with parsimonious dynamics by simultaneously learning a SINDy model for the dynamics of the intrinsic coordinates z. This regularization is achieved by constructing a library $\Theta \left(z\right)=\left[\theta_{1}\left(z\right),\theta_{2}\left(z\right),\ldots ,\theta_{p}\left(z\right)\right]$ of candidate basis functions, e.g., polynomials, and learning a sparse set of coefficients $\Xi =\left[\xi_{1},\ldots ,\xi_{d}\right]$ that defines the dynamical system

$$\frac{d}{dt}z\left(t\right)=g\left(z\left(t\right)\right)=\Theta \left(z\left(t\right)\right)\Xi .$$

While the library must be specified prior to training, the coefficients Ξ are learned with the NN parameters as part of the training procedure. Assuming derivatives $\dot{x}\left(t\right)$ of the original states are available or can be computed, one can calculate the derivative of the encoder variables as $\dot{z}\left(t\right)=\nabla_{x}\varphi \left(x\left(t\right)\right)\dot{x}\left(t\right)$ and enforce accurate prediction of the dynamics by incorporating the following term into the loss function:

$$L_{dz/dt}={∥\nabla_{x}\varphi \left(x\right)\dot{x}-\Theta \left(\varphi {\left(x\right)}^{T}\right)\Xi ∥}_{2}^{2}.$$ [3]

This term uses the SINDy model along with the gradient of the encoder to encourage the learned dynamical model to accurately predict the time derivatives of the encoder variables. We include an additional term in the loss function that ensures SINDy predictions can be used to reconstruct the time derivatives of the original data:

$$L_{dx/dt}={∥\dot{x}-\left(\nabla_{z}\psi \left(\varphi \left(x\right)\right)\right)\left(\Theta \left(\varphi {\left(x\right)}^{T}\right)\Xi \right)∥}_{2}^{2}.$$ [4]

We combine Eqs. **3** and **4** with the standard autoencoder loss

$$L_{\text{recon}}={∥x-\psi \left(\varphi \left(x\right)\right)∥}_{2}^{2},$$

which ensures that the autoencoder can accurately reconstruct the input data. We also include an $L_{1}$ regularization on the SINDy coefficients Ξ, which promotes sparsity of the coefficients and therefore encourages a parsimonious model for the dynamics. The combination of the above 4 terms gives the overall loss function

$$L_{\text{recon}}+\lambda_{1}L_{dx/dt}+\lambda_{2}L_{dz/dt}+\lambda_{3}L_{\text{reg}},$$

where the hyperparameters $\lambda_{1},\lambda_{2},\lambda_{3}$ determine the relative weighting of the 3 terms in the loss function.

In addition to the $L_{1}$ regularization, to obtain a model with only a few active terms, we also incorporate sequential thresholding into the training procedure as a proxy for $L_{0}$ sparsity (30). This technique is inspired by the original algorithm used for SINDy (8), which combined least-squares fitting with sequential thresholding to obtain a sparse model. To apply sequential thresholding during training, we specify a threshold that determines the minimum magnitude for coefficients in the SINDy model. At fixed intervals throughout the training, all coefficients below the threshold are set to zero and training resumes using only the terms left in the model. We train the network using the Adam optimizer (52). In addition to the loss function weightings and SINDy coefficient threshold, training requires the choice of several other hyperparameters including learning rate, number of intrinsic coordinates d, network size, and activation functions. Details of the training procedure are discussed in *SI Appendix*. Alternatively, one might attempt to learn the library functions using another neural network layer, a double sparse library (53), or kernel-based methods (54) for more flexible library representations.

## Results

**Fig. 2.**
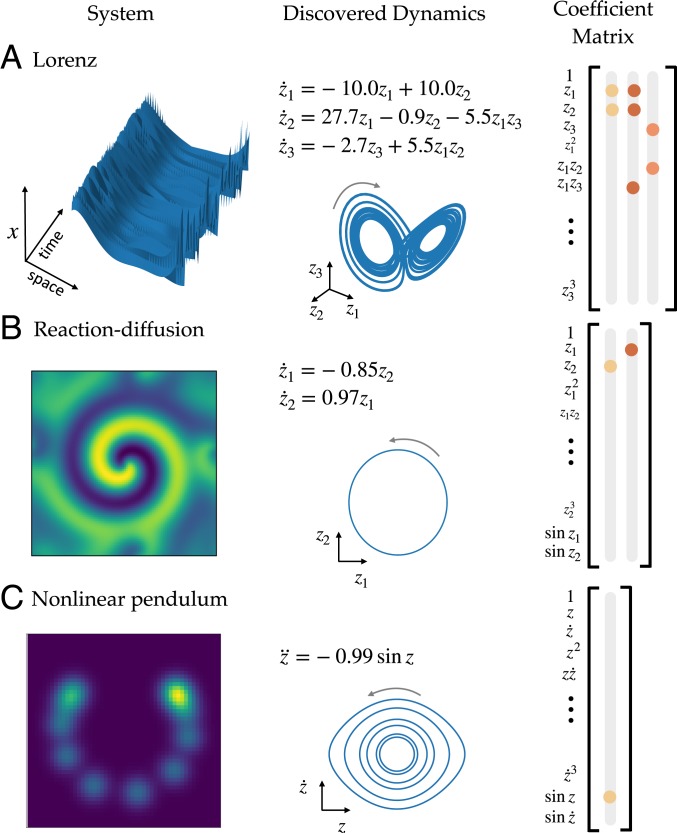
Discovered models for examples. (*A–C*) Equations, SINDy coefficients Ξ, and attractors for Lorenz (*A*), reaction–diffusion (*B*), and nonlinear pendulum (*C*) systems.

We demonstrate the success of the proposed method on 3 example systems: a high-dimensional system with the underlying dynamics generated from the canonical chaotic Lorenz system, a 2D reaction–diffusion system, and a 2D spatial representation (synthetic video) of the nonlinear pendulum. Results are shown in Fig. 2.

### Chaotic Lorenz System.

We first construct a high-dimensional example problem with dynamics based on the chaotic Lorenz system. The Lorenz system is a canonical model used as a test case, with dynamics given by the following equations:

$${ż}_{1}=\sigma \left(z_{2}-z_{1}\right)$$ [5a]

$${ż}_{2}=z_{1}\left(\rho -z_{3}\right)-z_{2}$$ [5b]

$${ż}_{3}=z_{1}z_{2}-\beta z_{3}.$$ [5c]

The dynamics of the Lorenz system are chaotic and highly nonlinear, making it an ideal test problem for model discovery. To create a high-dimensional dataset based on this system, we choose 6 fixed spatial modes $u_{1},\ldots ,u_{6}\in R^{128}$, given by Legendre polynomials, and define

$$\begin{array}{ll} x\left(t\right) & =u_{1}z_{1}\left(t\right)+u_{2}z_{2}\left(t\right)+u_{3}z_{3}\left(t\right)+u_{4}z_{1}{\left(t\right)}^{3}+u_{5}z_{2}{\left(t\right)}^{3}\\ & \;+u_{6}z_{3}{\left(t\right)}^{3}. \end{array}$$ [6]

**Fig. 3.**
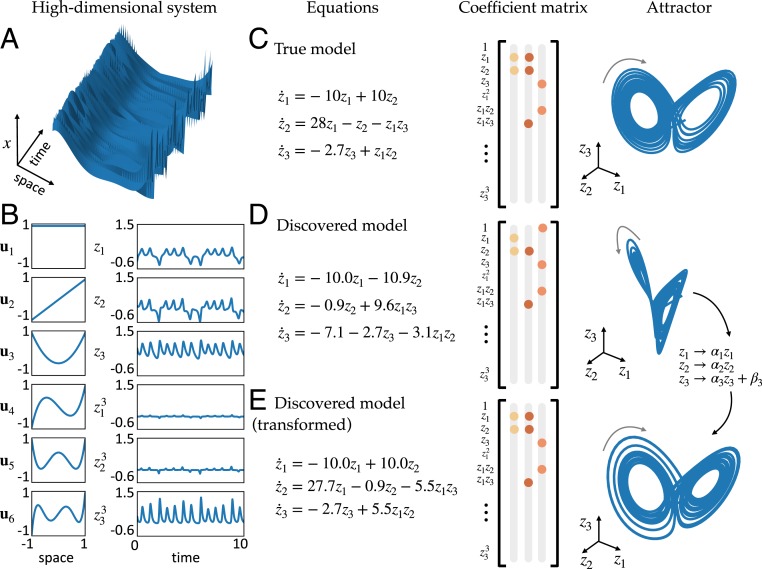
Model results on the high-dimensional Lorenz example. (*A*) Trajectories of the chaotic Lorenz system ($z\left(t\right)\in R^{3}$) are used to create a high-dimensional dataset ($x\left(t\right)\in R^{128}$). (*B*) The spatial modes are created from the first 6 Legendre polynomials and the temporal modes are the variables in the Lorenz system and their cubes. The spatial and temporal modes are combined to create the high-dimensional dataset via **[6]**. (*C* and *D*) The equations, SINDy coefficients Ξ, and attractors for the original Lorenz system and a dynamical system discovered by the SINDy autoencoder. The attractors are constructed by simulating the dynamical system forward in time from a single initial condition. (*E*) Applying a suitable variable transformation to the system in *D* reveals a model with the same sparsity pattern as the original Lorenz system. The parameters are close in value to the original system, with the exception of an arbitrary scaling, and the attractor has a similar structure to the original system.

This results in a dataset that is a nonlinear combination of the true Lorenz variables, shown in Fig. 3*A*. The spatial and temporal modes that combine to give the full dynamics are shown in Fig. 3*B*. Full details of how the dataset is generated are given in *SI Appendix*.

Fig. 3*D* shows the dynamical system discovered by the SINDy autoencoder. While the resulting model does not appear to match the original Lorenz system, the discovered model is parsimonious, with only 7 active terms, and the dynamics exhibit an attractor with a 2-lobe structure, similar to that of the original Lorenz attractor. Additionally, by choosing a suitable variable transformation the discovered model can be rewritten in the same form as the original Lorenz system. This demonstrates that the SINDy autoencoder is able to recover the correct sparsity pattern of the dynamics. The coefficients of the discovered model are close to the original parameters of the Lorenz system, up to an arbitrary scaling, which accounts for the difference in magnitude of the coefficients of $z_{1}z_{3}$ in the second equation and $z_{1}z_{2}$ in the third equation.

On test trajectories from 100 initial conditions sampled from the training distribution, the relative $L_{2}$ errors in predicting x, $\dot{x}$, and $\dot{z}$ are $3\times 10^{-5}$, $2\times 10^{-4}$, and $7\times 10^{-4}$, respectively. For initial conditions outside of the training distribution, the model has higher relative $L_{2}$ errors on 100 test trajectories of 0.016, 0.126, and 0.078 for x, $\dot{x}$, and $\dot{z}$. In both cases, the resulting SINDy models produce dynamics that are qualitatively similar to the true trajectories, although due to the chaotic nature of the Lorenz system and its sensitivity to parameters and initial conditions, the phase of most predicted trajectories diverges from the true trajectories after a short period. Improved prediction over a longer duration may be achieved by increased parameter refinement or training with longer trajectories.

### Reaction–Diffusion.

In practice, many high-dimensional datasets of interest come from dynamics governed by PDEs with more complicated interactions between spatial and temporal dynamics. To test the method on data generated by a PDE, we consider a lambda–omega reaction–diffusion system governed by

$$\begin{array}{ll} u_{t} & =\left(1-\left(u^{2}+v^{2}\right)\right)u+\beta \left(u^{2}+v^{2}\right)v+d_{1}\left(u_{xx}+u_{yy}\right)\\ v_{t} & =-\beta \left(u^{2}+v^{2}\right)u+\left(1-\left(u^{2}+v^{2}\right)\right)v+d_{2}\left(v_{xx}+v_{yy}\right) \end{array}$$

with $d_{1},d_{2}=0.1$ and β=1. This set of equations generates a spiral wave formation, whose behavior can be approximately captured by 2 oscillating spatial modes. We apply our method to snapshots of u(x,y,t) generated by the above equations. Snapshots are collected at discretized points of the xy domain, resulting in a high-dimensional input dataset with $n=10^{4}$.

We train the SINDy autoencoder with d=2. The resulting model is shown in Fig. 2*B*. The network discovers a model with nonlinear oscillatory dynamics. On test data, the relative $L_{2}$ error for the input data x and the input derivatives $\dot{x}$ is 0.016. The relative $L_{2}$ error for $\dot{z}$ is 0.002. Simulation of the dynamical model accurately captures the low-dimensional dynamics, with relative $L_{2}$ error of z totaling $1\times 10^{-4}$.

### Nonlinear Pendulum.

As a final example, we consider a simulated video of a nonlinear pendulum. The nonlinear pendulum is governed by the following second-order differential equation:

$$\ddot{z}=-\sin z.$$

We simulate the system from several initial conditions and generate a series of snapshot images with a 2D Gaussian centered at the center of mass, determined by the pendulum’s angle z. This series of images is the high-dimensional data input to the autoencoder. Despite the fact that the position of the pendulum can be represented by a simple 1-dimensional variable, methods such as PCA are unable to obtain a low-dimensional representation of this dataset. A nonlinear autoencoder, however, is able to discover a 1-dimensional representation of the dataset.

For this example, we use a second-order SINDy model with a library of functions including the first derivatives $\dot{z}$ to predict the second derivative $\ddot{z}$. This approach is the same as with a first-order SINDy model but requires estimates of the second derivatives as well. Second-order gradients of the encoder and decoder are therefore also required. Computation of the derivatives is discussed in *SI Appendix*.

The SINDy autoencoder is trained with d=1. Of the 10 training instances, 5 correctly identify the nonlinear pendulum equation. We calculate test error on trajectories from 50 randomly chosen initial conditions sampled from the same distribution as the training data. The best model has a relative $L_{2}$ error of $8\times 10^{-4}$ for the decoder reconstruction of the input x. The relative $L_{2}$ errors of the SINDy model predictions for $\ddot{x}$ and $\ddot{z}$ are $3\times 10^{-4}$ and $2\times 10^{-2}$, respectively.

## Discussion

We have presented a data-driven method for discovering interpretable, low-dimensional dynamical models and their associated coordinates from high-dimensional data. The simultaneous discovery of both is critical for generating dynamical models that are sparse and hence interpretable. Our approach takes advantage of the power of NNs by using a flexible autoencoder architecture to discover nonlinear coordinate transformations that enable the discovery of parsimonious, nonlinear governing equations. This work addresses a major limitation of prior approaches for model discovery, which is that the proper choice of measurement coordinates is often unknown. We demonstrate this method on 3 example systems, showing that it is able to identify coordinates associated with parsimonious dynamical equations. Our code is publicly available at http://github.com/kpchamp/SindyAutoencoders (55).

A current limitation of our approach is the requirement for clean measurement data that are approximately noise-free. Fitting a continuous-time dynamical system with SINDy requires reasonable estimates of the derivatives, which may be difficult to obtain from noisy data. While this represents a challenge, approaches for estimating derivatives from noisy data such as the total variation regularized derivative can prove useful in providing derivative estimates (56). Moreover, there are emerging NN architectures explicitly constructed for separating signals from noise (57), which can be used as a preprocessing step in the data-driven discovery process advocated here. Alternatively our method can be used to fit a discrete-time dynamical system, in which case derivative estimates are not required. It is also possible to use the integral formulation of SINDy to abate noise sensitivity (39).

A major problem with deep-learning approaches is that models are typically neither interpretable nor generalizable. Specifically, NNs trained solely for prediction may fail to generalize to classes of behaviors not seen in the training set. We have demonstrated an approach for using NNs to obtain classically interpretable models through the discovery of low-dimensional dynamical systems, which are well studied and often have physical interpretations. While the autoencoder network still has the same limited interpretability and generalizability as other NNs, the dynamical model has the potential to generalize to other parameter regimes of the dynamics. Although the coordinate transformation learned by the autoencoder may not generalize to data regimes far from the original training set, if the dynamics are known, the autoencoder can be retrained on new data with fixed terms in the latent dynamics space (see *SI Appendix* for discussion). The problem of relearning a coordinate transformation for a system with known dynamics is simplified from the original challenge of learning the correct form of the underlying dynamics without knowledge of the proper coordinate transformation.

The challenge of utilizing NNs to answer scientific questions requires careful consideration of their strengths and limitations. While advances in deep learning and computing power present a tremendous opportunity for new scientific breakthroughs, care must be taken to ensure that valid conclusions are drawn from the results. One promising strategy is to combine machine-learning approaches with well-established domain knowledge: For instance, physics-informed learning leverages physical assumptions into NN architectures and training methods. Methods that provide interpretable models have the potential to enable new discoveries in data-rich fields. This work introduced a flexible framework for using NNs to discover models that are interpretable from a standard dynamical systems perspective. While this formulation used an autoencoder to achieve full state reconstruction, similar architectures could be used to discover embeddings that satisfy alternative conditions. In the future, this approach could be adapted using domain knowledge to discover new models in specific fields.

## Supplementary Material

Supplementary File
