# Supplementary material for: Data-driven discovery of coordinates and governing equations
Source: Proc Natl Acad Sci U S A. 2019 Oct 21;116(45):22445–51. doi: 10.1073/pnas.1906995116 (PMC6842598; doi:10.1073/pnas.1906995116)
Supplement: Supplementary File [file pnas.1906995116.sapp.pdf]

# Supplementary Information for

## Data-driven discovery of coordinates and governing equations

**Kathleen Champion, Bethany Lusch, J. Nathan Kutz, and Steven L. Brunton**

**Kathleen Champion.**
**E-mail: kpchamp@uw.edu**

### This PDF file includes:

Supplementary text
Figs. S1 to S5
Tables S1 to S3
References for SI reference citations


www.pnas.org/cgi/doi/10.1073/pnas.1906995116

## Supporting Information Text

## Network Architecture and Training

**Network architecture.** The autoencoder network consists of a series of fully-connected layers. Each layer has an associated weight matrix $\mathbf{W}$ and bias vector $\mathbf{b}$. We use sigmoid activation functions $f(x) = 1/(1+\exp(-x))$, which are applied at all layers of the network, except for the last layer of the encoder and the last layer of the decoder. Other choices of activation function, such as rectified linear units and exponential linear units, may also be used and appear to achieve similar results.

**Loss function.** The loss function used in training is a weighted sum of four terms: autoencoder reconstruction $\mathcal{L}_{\text{recon}}$, SINDy prediction on the input variables $\mathcal{L}_{d\mathbf{x}/dt}$, SINDy prediction on the encoder variables $\mathcal{L}_{d\mathbf{z}/dt}$, and SINDy coefficient regularization $\mathcal{L}_{\text{reg}}$. For a data set with $m$ input samples, each loss is explicitly defined as follows:

$$\begin{aligned}
\mathcal{L}_{\text{recon}} &= \frac{1}{m}\sum_{i=1}^{m} \|\mathbf{x}_i - \psi(\varphi(\mathbf{x}_i))\|_2^2 \\
\mathcal{L}_{d\mathbf{x}/dt} &= \frac{1}{m}\sum_{i=1}^{m} \left\|\dot{\mathbf{x}}_i - \left(\nabla_{\mathbf{z}}\psi(\varphi(\mathbf{x}_i))\right)\left(\boldsymbol{\Theta}(\varphi(\mathbf{x}_i)^T)\boldsymbol{\Xi}\right)\right\|_2^2 \\
\mathcal{L}_{d\mathbf{z}/dt} &= \frac{1}{m}\sum_{i=1}^{m} \left\|\nabla_{\mathbf{x}}\varphi(\mathbf{x}_i)\dot{\mathbf{x}}_i - \boldsymbol{\Theta}(\varphi(\mathbf{x}_i)^T)\boldsymbol{\Xi}\right\|_2^2 \\
\mathcal{L}_{\text{reg}} &= \frac{1}{pd}\|\boldsymbol{\Xi}\|_1 .
\end{aligned}$$

The total loss function is

$$\mathcal{L}_{\text{recon}} + \lambda_1\mathcal{L}_{d\mathbf{x}/dt} + \lambda_2\mathcal{L}_{d\mathbf{z}/dt} + \lambda_3\mathcal{L}_{\text{reg}}.$$

$\mathcal{L}_{\text{recon}}$ ensures that the autoencoder can accurately reconstruct the data from the intrinsic coordinates. $\mathcal{L}_{d\mathbf{x}/dt}$ and $\mathcal{L}_{d\mathbf{z}/dt}$ ensure that the discovered SINDy model captures the dynamics of the system by ensuring that the model can predict the derivatives from the data. $\mathcal{L}_{\text{reg}}$ promotes sparsity of the coefficients in the SINDy model.

**Computing derivatives.** Computing the derivatives of the encoder variables requires propagating derivatives through the network. Our network makes use of an activation function $f(\cdot)$ that operates elementwise. Given an input $\mathbf{x}$, we define the pre-activation values at the $j$th encoder layer as

$$\mathbf{l}_j = f(\mathbf{l}_{j-1})\mathbf{W}_j + \mathbf{b}_j.$$

The first layer applies the weights and biases directly to the input so that

$$\mathbf{l}_0 = \mathbf{x}\mathbf{W}_0 + \mathbf{b}_0.$$

The activation function is not applied to the last layer, so for an encoder with $L$ hidden layers the autoencoder variables are defined as

$$\mathbf{z} = f(\mathbf{l}_{L-1})\mathbf{W}_L + \mathbf{b}_L.$$

Assuming that derivatives $d\mathbf{x}/dt$ are available or can be computed, derivatives $d\mathbf{z}/dt$ can also be computed:

$$\frac{d\mathbf{z}}{dt} = \left(f'(\mathbf{l}_{L-1}) \circ \frac{d\mathbf{l}_{L-1}}{dt}\right)\mathbf{W}_L$$

with

$$\begin{aligned}
\frac{d\mathbf{l}_j}{dt} &= \left(f'(\mathbf{l}_{j-1}) \circ \frac{d\mathbf{l}_{j-1}}{dt}\right)\mathbf{W}_j \\
\frac{d\mathbf{l}_0}{dt} &= \frac{d\mathbf{x}}{dt}\mathbf{W}_0.
\end{aligned}$$

For the nonlinear pendulum example, we use a second order SINDy model that requires the calculation of second derivatives. Second derivatives can be computed using the following:

$$\begin{aligned}
\frac{d^2\mathbf{z}}{dt^2} &= \left(f''(\mathbf{l}_{L-1}) \circ \frac{d\mathbf{l}_{L-1}}{dt} \circ \frac{d\mathbf{l}_{L-1}}{dt} + f'(\mathbf{l}_{L-1}) \circ \frac{d^2\mathbf{l}_{L-1}}{dt^2}\right)\mathbf{W}_L \\
\frac{d^2\mathbf{l}_j}{dt^2} &= \left(f''(\mathbf{l}_{j-1}) \circ \frac{d\mathbf{l}_{j-1}}{dt} \circ \frac{d\mathbf{l}_{j-1}}{dt} + f'(\mathbf{l}_{j-1}) \circ \frac{d^2\mathbf{l}_{j-1}}{dt^2}\right)\mathbf{W}_j \\
\frac{d\mathbf{l}_0}{dt} &= \frac{d^2\mathbf{x}}{dt^2}\mathbf{W}_0.
\end{aligned}$$

**Training procedure.** We train multiple models for each of the example systems. Each instance of training has a different random initialization of the network weights. The weight matrices $\mathbf{W}_j$ are initialized using the Xavier initialization: the entries are chosen from a random uniform distribution over $[-\sqrt{6/\alpha}, \sqrt{6/\alpha}]$ where $\alpha$ is the dimension of the input plus the dimension of the output (1). The bias vectors $\mathbf{b}_j$ are initialized to 0 and the SINDy model coefficients $\boldsymbol{\Xi}$ are initialized so that every entry is 1. We train each model using the Adam optimizer for a fixed number of epochs (2). The learning rate and number of training epochs used are specific to each example.

To obtain parsimonious dynamical models, we use a sequential thresholding procedure that promotes sparsity on the coefficients in $\boldsymbol{\Xi}$, which represent the dynamics on the latent variables $\mathbf{z}$. Every 500 epochs, we set all coefficients in $\boldsymbol{\Xi}$ with a magnitude of less than 0.1 to 0, effectively removing these terms from the SINDy model. This is achieved by using a mask $\boldsymbol{\Upsilon}$, consisting of 1s and 0s, that determines which terms remain in the SINDy model. Thus the true SINDy terms in the loss function are given by

$$\mathcal{L}_{d\mathbf{x}/dt} = \frac{1}{m}\sum_{i=1}^{m} \left\| \dot{\mathbf{x}}_i - (\nabla_{\mathbf{z}}\psi(\varphi(\mathbf{x}_i))) \left(\boldsymbol{\Theta}(\varphi(\mathbf{x}_i)^T)(\boldsymbol{\Upsilon} \circ \boldsymbol{\Xi})\right) \right\|_2^2$$

$$\mathcal{L}_{d\mathbf{z}/dt} = \frac{1}{m}\sum_{i=1}^{m} \left\| \nabla_{\mathbf{x}}\varphi(\mathbf{x}_i)\dot{\mathbf{x}}_i - \boldsymbol{\Theta}(\varphi(\mathbf{x}_i)^T)(\boldsymbol{\Upsilon} \circ \boldsymbol{\Xi}) \right\|_2^2$$

where $\boldsymbol{\Upsilon}$ is passed in separately and not updated by the optimization algorithm. Once a term has been thresholded out during training, it is permanently removed from the SINDy model. Therefore the number of active terms in the SINDy model can only be decreased as training continues. The $L_1$ regularization on $\boldsymbol{\Xi}$ encourages the model coefficients to decrease in magnitude, which combined with the sequential thresholding produces a parsimonious dynamical model.

While the $L_1$ regularization penalty on $\boldsymbol{\Xi}$ promotes sparsity in the resulting SINDy model, it also encourages nonzero terms to have smaller magnitudes. This results in a trade-off between accurately reconstructing the dynamics of the system and reducing the magnitude of the SINDy coefficients, where the trade-off is determined by the relative magnitudes of the loss weight penalties $\lambda_1, \lambda_2$ and the regularization penalty $\lambda_3$. The specified training procedure therefore typically results in models with coefficients that are slightly smaller in magnitude than those which would best reproduce the dynamics. To account for this, we add an additional coefficient refinement period to the training procedure. To perform this refinement, we lock in the sparsity pattern in the dynamics by fixing the coefficient mask $\boldsymbol{\Upsilon}$ and continue training for 1000 epochs without the $L_1$ regularization on $\boldsymbol{\Xi}$. This ensures that the best coefficients are found for the resulting SINDy model and also allows the training procedure to refine the encoder and decoder parameters. This procedure is analogous to running a debiased regression following the use of LASSO to select model terms (3).

**Choice of hyperparameters.** The training procedure described above requires the choice of several hyperparameters: chiefly the number of intrinsic coordinates $d$ in the middle layer of the autoencoder and the loss weight penalties $\lambda_1, \lambda_2, \lambda_3$. The choice of these parameters greatly impacts the success of the training procedure. Here we outline some guidelines for choosing these parameters.

The most important hyperparameter choice is the number of intrinsic coordinates $d$, as this impacts the interpretation of the reduced space and the associated dynamical model. In the examples discussed here we had a good sense of how many coordinates were necessary, but for many systems the choice may not be obvious. To choose $d$, we suggest first training a standard autoencoder without the associated SINDy model to determine the minimum number of coordinates necessary to reproduce the input data. As simpler models (lower $d$) are typically easier to interpret, one should start by using the smallest $d$ possible. Once this minimum has been found, the full SINDy autoencoder can be trained. It is possible that more coordinates may be needed to capture the dynamics, in which case the method may accurately reproduce the input but fail to find a good model for the dynamics. In this case $d$ could be increased from the minimum until a suitable dynamical model is found. Choosing $d$ that is greater than necessary may still result in a valid model for the dynamics; however, obtaining a sparse model may be more difficult.

The choice of loss weight penalties $\lambda_1, \lambda_2, \lambda_3$ also has a significant impact on the success of the training procedure. The parameter $\lambda_1$ determines the importance of the SINDy prediction in the original input space. We generally choose $\lambda_1$ to be slightly less than the ratio $\sum_{i=1}^{m} \|\mathbf{x}_i\|_2^2 / \sum_{i=1}^{m} \|\dot{\mathbf{x}}_i\|_2^2$. This slightly prioritizes the reconstruction of $\mathbf{x}$ over prediction of $\dot{\mathbf{x}}$ in the training. This is important to ensure that the autoencoder weights are being trained to reproduce $\mathbf{x}$ and that it is the SINDy model that gives an accurate prediction of $\dot{\mathbf{x}}$. We choose $\lambda_2$ to be one or two orders of magnitude less than $\lambda_1$. If $\lambda_2$ is too large, it encourages shrinking of the magnitude of $\dot{\mathbf{z}}$ to minimize $\mathcal{L}_{d\mathbf{z}/dt}$; however, having $\lambda_2$ nonzero encourages a good prediction by the SINDy model in the $\dot{\mathbf{z}}$ space. The third parameter $\lambda_3$ determines the strength of the regularization of the SINDy model coefficients $\boldsymbol{\Xi}$ and thus affects the sparsity of the resulting models. If $\lambda_3$ is too large, the model will be too simple and achieve poor prediction; if it is too small, the models will be nonsparse and prone to overfitting. This loss weight requires the most tuning and should typically be chosen last by trying a range of values and assessing the level of sparsity in the resulting model.

In addition to the hyperparameters used in the neural network training, this method requires a choice of library functions for the SINDy model. The examples shown here use polynomial library terms, along with sine terms in the latter two examples. For general systems, the best library functions to use may be unknown, and choosing the wrong library functions can obscure the simplest model. A recommended practice is to start with polynomial models, as many common physical models contain polynomials representing dominant balance terms or are a coordinate transformation away from a normal form characterized

by a few polynomial nonlinearities. Polynomials can also represent Taylor series approximations for a broad class of smooth functions. For some systems, the choice of library functions could be informed by application specific knowledge. Alternatively, one might attempt to learn the library functions, for example, using another neural network layer; however, this approach would generally hamper the interpretability and generalizability of the resulting dynamical model. Other approaches, such as kernel-based methods, also have the potential to enable more flexible library representations (4).

**Model selection.** Random initialization of the NN weights is standard practice for deep learning approaches. This results in the discovery of different models for different instances of training, which necessitates comparison among multiple models. In this work, when considering the success of a resulting model, one must consider the parsimony of the SINDy model, how well the decoder reconstructs the input, and how well the SINDy model captures the dynamics.

To assess model performance, we calculate the relative $L_2$ error of both the input data $\mathbf{x}$ and its derivative $\dot{\mathbf{x}}$:

$$e_{\mathbf{x}} = \frac{\sum_{i=1}^{m} \|\mathbf{x}_i - \psi(\varphi(\mathbf{x}_i))\|_2^2}{\sum_{i=1}^{m} \|\mathbf{x}_i\|_2^2}, \quad e_{\dot{\mathbf{x}}} = \frac{\sum_{i=1}^{m} \left\|\dot{\mathbf{x}}_i - (\nabla_{\mathbf{z}}\psi(\varphi(\mathbf{x}_i)))\left(\mathbf{\Theta}(\varphi(\mathbf{x}_i)^T)\mathbf{\Xi}\right)\right\|_2^2}{\sum_{i=1}^{m} \|\dot{\mathbf{x}}_i\|_2^2}. \qquad [1]$$

These error values capture the decoder reconstruction and the fit of the dynamics, respectively. When considering parsimony, we consider the number of active terms in the resulting SINDy model. While parsimonious models are desirable for ease of analysis and interpretability, a model that is too parsimonious may be unable to fully capture the dynamics. In general, for the examples explored, we find that models with fewer active terms perform better on validation data (lower relative $L_2$ error $e_{\dot{\mathbf{x}}}$) whereas models with more active terms tend to overfit the training data. In reporting our results, we also calculate the relative $L_2$ error in predicting $\dot{\mathbf{z}}$:

$$e_{\dot{\mathbf{z}}} = \frac{\sum_{i=1}^{m} \left\|\nabla_{\mathbf{x}}\varphi(\mathbf{x}_i)\dot{\mathbf{x}}_i - \mathbf{\Theta}(\varphi(\mathbf{x}_i)^T)(\mathbf{\Upsilon} \circ \mathbf{\Xi})\right\|_2^2}{\sum_{i=1}^{m} \|\nabla_{\mathbf{x}}\varphi(\mathbf{x}_i)\dot{\mathbf{x}}_i\|_2^2}.$$

For each example system, we apply the training procedure to ten different initializations of the network and compare the resulting models. For the purpose of demonstration, for each example we show results for a chosen "best" model, which is taken to be the model with the lowest relative $L_2$ error on validation data among models with the fewest active coefficients. While every instance of training does not result in the exact same SINDy sparsity pattern, the network tends to discover a few different closely related forms of the dynamics.

**Code.** We use the Python API for TensorFlow to implement and train our network (5). Our code is publicly available at github.com/kpchamp/SindyAutoencoders.

## Example Systems

**Chaotic Lorenz system.** To create a high-dimensional data set with dynamics defined by the Lorenz system, we choose six spatial modes $\mathbf{u}_1, \ldots, \mathbf{u}_6 \in \mathbb{R}^{128}$ and take

$$\mathbf{x}(t) = \mathbf{u}_1 z_1(t) + \mathbf{u}_2 z_2(t) + \mathbf{u}_3 z_3(t) + \mathbf{u}_4 z_1(t)^3 + \mathbf{u}_5 z_2(t)^3 + \mathbf{u}_6 z_3(t)^3.$$

where the dynamics of $\mathbf{z}$ are specified by the Lorenz equations

$$\begin{aligned}\dot{z}_1 &= \sigma(z_2 - z_1)\\ \dot{z}_2 &= z_1(\rho - z_3) - z_2\\ \dot{z}_3 &= z_1 z_2 - \beta z_3\end{aligned}$$

with standard parameter values of $\sigma = 10, \rho = 28, \beta = 8/3$. We choose our spatial modes $\mathbf{u}_1, \ldots, \mathbf{u}_6$ to be the first six Legendre polynomials defined at 128 grid points on a 1D spatial domain $[-1, 1]$. To generate our data set, we simulate the system from 2048 initial conditions for the training set, 20 for the validation set, and 100 for the test set. For each initial condition we integrate the system forward in time from $t = 0$ to $t = 5$ with a spacing of $\Delta t = 0.02$ to obtain 250 samples. Initial conditions are chosen randomly from a uniform distribution over $z_1 \in [-36, 36]$, $z_2 \in [-48, 48]$, $z_3 \in [-16, 66]$. This results in a training set with 512,000 total samples.

Following the training procedure described above, we learn ten models using the single set of training data (variability among the models comes from the initialization of the network weights). The hyperparameters used for training are shown in Table S1. For each model we run the training procedure for $10^4$ epochs, followed by a refinement period of $10^3$ epochs. Of the ten models, two have 7 active terms, two have 10 active terms, one has 11 active terms, and five have 15 or more active terms. While all models have $e_{\mathbf{x}}, e_{\dot{\mathbf{x}}} < 0.01$, the three models with 20 or more active terms have the worst performance predicting $\dot{\mathbf{x}}$. The two models with 10 active terms have the lowest overall error, followed by models with 7, 15, and 18 active terms. While the models with 10 active terms have a lower overall error than the models with 7 terms, both have a very low error and thus we choose to highlight the model with the fewest active terms. A model with 10 active terms is shown in Figure S1 for comparison.

For analysis, we highlight the model with the lowest error among the models with the fewest active terms. The discovered model has equations

$$\dot{z}_1 = -10.0z_1 - 10.9z_2$$
$$\dot{z}_2 = -0.9z_2 + 9.6z_1z_3$$
$$\dot{z}_3 = -7.1 - 2.7z_3 - 3.1z_1z_2.$$

While the structure of this model appears to be different from that of the original Lorenz system, we can define an affine transformation that gives it the same structure. The variable transformation $z_1 = \alpha_1\tilde{z}_1$, $z_2 = \alpha_2\tilde{z}_2$, $z_3 = \alpha_3\tilde{z}_3 + \beta_3$ gives the following transformed system of equations:

$$\begin{aligned}\dot{\tilde{z}}_1 &= \frac{1}{\alpha_1}\left(-10.0\alpha_1\tilde{z}_1 - 10.9\alpha_2\tilde{z}_2\right) \\ &= -10.0\tilde{z}_1 - 10.9\frac{\alpha_2}{\alpha_1}\tilde{z}_2 \end{aligned} \quad [2a]$$

$$\begin{aligned}\dot{\tilde{z}}_2 &= \frac{1}{\alpha_2}\left(-0.9\alpha_2\tilde{z}_2 + 9.6\alpha_1\tilde{z}_1(\alpha_3\tilde{z}_3 + \beta_3)\right) \\ &= 9.6\frac{\alpha_1}{\alpha_2}\beta_3\tilde{z}_1 - 0.9\tilde{z}_2 + 9.6\frac{\alpha_1\alpha_3}{\alpha_2}\tilde{z}_1\tilde{z}_3 \end{aligned} \quad [2b]$$

$$\begin{aligned}\dot{\tilde{z}}_3 &= \frac{1}{\alpha_3}\left(-7.1 - 2.7(\alpha_3\tilde{z}_3 + \beta_3) - 3.1\alpha_1\alpha_2\tilde{z}_1\tilde{z}_2\right) \\ &= \frac{1}{\alpha_3}(-7.1 - 2.7\beta_3) - 2.7\tilde{z}_3 - 3.1\frac{\alpha_1\alpha_2}{\alpha_3}\tilde{z}_1\tilde{z}_2. \end{aligned} \quad [2c]$$

By choosing $\alpha_1 = 1$, $\alpha_2 = -0.917$, $\alpha_3 = 0.524$, $\beta_3 = -2.665$, the system becomes

$$\dot{\tilde{z}}_1 = -10.0\tilde{z}_1 + 10.0\tilde{z}_2 \quad [3a]$$
$$\dot{\tilde{z}}_2 = 27.7\tilde{z}_1 - 0.9\tilde{z}_2 - 5.5\tilde{z}_1\tilde{z}_3 \quad [3b]$$
$$\dot{\tilde{z}}_3 = -2.7\tilde{z}_3 + 5.5\tilde{z}_1\tilde{z}_2. \quad [3c]$$

This has the same form as the original Lorenz equations with parameters that are close in value, apart from an arbitrary scaling that affects the magnitude of the coefficients of $\tilde{z}_1\tilde{z}_3$ in Eq. (3b) and $\tilde{z}_1\tilde{z}_2$ in Eq. (3c). The attractor dynamics for this system are very similar to the original Lorenz attractor and are shown in Figure 3c in the main text.

To evaluate performance, we report the relative $L_2$ error of predicting $\mathbf{x}$, $\dot{\mathbf{x}}$, and $\dot{\mathbf{z}}$, as defined in Eq. (1). We look at performance on two different test sets: one created from initial conditions sampled from the same distribution as the training data, and one from initial conditions outside this distribution. Both test sets consist of trajectories from 100 initial conditions, simulated from $t = 0$ to 5 (as in the training set). The initial conditions in the training set were sampled from a uniform distribution in a box surrounding the Lorenz attractor. To generate test initial conditions from outside the training distribution, we expand the size of this box by 50% in each dimension and create a test set of 100 randomly sampled initial conditions (ensuring that all initial conditions are outside of the training distribution). For the in-distribution initial conditions, the relative $L_2$ errors in predicting $\mathbf{x}$, $\dot{\mathbf{x}}$, and $\dot{\mathbf{z}}$ are $3 \times 10^{-5}$, $2 \times 10^{-4}$, and $7 \times 10^{-4}$, respectively. For the initial conditions outside of the training distribution, the relative $L_2$ errors are 0.016, 0.126, and 0.078 for $\mathbf{x}$, $\dot{\mathbf{x}}$, and $\dot{\mathbf{z}}$, respectively.

The learning procedure discovers a dynamical model by fitting coefficients that predict the continuous-time derivatives of the variables in a dynamical system. Thus it is possible for the training procedure to discover a model with unstable dynamics or which is unable to predict the true dynamics through simulation. We assess the validity of the discovered model by simulating the dynamics of the discovered low-dimensional dynamical system. Simulation of the system shows that the system is stable with trajectories existing on an attractor very similar to the original Lorenz attractor. Additionally, the discovered system is able to predict the dynamics in the reduced space. The fourth panel in Figure S1a shows the trajectories found by stepping the discovered model forward in time as compared with the values of $\mathbf{z}$ obtained by mapping samples of the high-dimensional data through the encoder. The trajectories match closely up to around $t = 5$, which is the duration of trajectories contained in the training set. After that the trajectories diverge, but the predicted trajectories remain on an attractor. The Lorenz dynamics are chaotic, and thus slight differences in coefficients or initial conditions cause trajectories to diverge quickly. Figure S2 and Figure S3 each show simulated trajectories from nine additional initial conditions, sampled from within the training distribution and outside of the training distribution, respectively. While the simulations for initial conditions outside of the training distribution diverge from the true trajectories more quickly than for initial conditions within the training distribution, both largely capture the qualitative behaviors of the dynamics. In several cases, they remain close to the true trajectories for several time units.

For comparison, in Figure S1b we show a second model discovered by the training procedure. This model has 10 active terms, as compared with 7 in the true Lorenz system. While the model contains additional terms not present in the original system, the dynamics lie on an attractor with a similar two lobe structure. Additionally, the system is able to predict the dynamics through simulation. This model has a lower error on test data than the original 7 term model, with relative $L_2$ errors $e_{\mathbf{x}} \approx 2 \times 10^{-6}$, $e_{\dot{\mathbf{x}}} \approx 6 \times 10^{-5}$, and $e_{\dot{\mathbf{z}}} \approx 3 \times 10^{-4}$ for initial conditions in the same distribution as the training data. For a set of 100 test initial conditions sampled from outside of the training distribution (chosen from the same distribution as used for the 7 term model), the relative $L_2$ errors are $e_{\mathbf{x}} \approx 0.021$, $e_{\dot{\mathbf{x}}} \approx 0.154$, and $e_{\dot{\mathbf{z}}} \approx 0.102$.

**Reaction-diffusion.** We generate data from a high-dimensional lambda-omega reaction-diffusion system governed by

$$u_t = (1 - (u^2 + v^2))u + \beta(u^2 + v^2)v + d_1(u_{xx} + u_{yy})$$
$$v_t = -\beta(u^2 + v^2)u + (1 - (u^2 + v^2))v + d_2(v_{xx} + v_{yy})$$

with $d_1, d_2 = 0.1$ and $\beta = 1$. The system is simulated from a single initial condition from $t = 0$ to $t = 10$ with a spacing of $\Delta t = 0.05$ for a total of 10,000 samples. The initial condition is defined as

$$u(y_1, y_2, 0) = \tanh\left(\sqrt{y_1^2 + y_2^2}\cos\left(\angle(y_1 + iy_2) - \sqrt{y_1^2 + y_2^2}\right)\right)$$
$$v(y_1, y_2, 0) = \tanh\left(\sqrt{y_1^2 + y_2^2}\sin\left(\angle(y_1 + iy_2) - \sqrt{y_1^2 + y_2^2}\right)\right)$$

over a spatial domain of $y_1 \in [-10, 10],\ y_2 \in [-10, 10]$ discretized on a grid with 100 points on each spatial axis. The solution of these equations results in a spiral wave formation. We apply our method to snapshots of $u(y_1, y_2, t)$ generated by the above equations, multiplied by a Gaussian $f(y_1, y_2) = \exp(-0.1(y_1^2 + y_2^2))$ centered at the origin to localize the spiral wave in the center of the domain. Our input data is thus defined as $\mathbf{x}(t) = f(:,:) \circ u(:,:,t) \in \mathbb{R}^{10^4}$. We also add Gaussian noise with a standard deviation of $10^{-6}$ to both $\mathbf{x}$ and $\dot{\mathbf{x}}$. Four time snapshots of the input data are shown in Figure S4a.

We divide the total number of samples into training, validation, and test sets: the last 1000 samples are taken as the test set, 1000 samples are chosen randomly from the first 9000 samples as a validation set, and the remaining 8000 samples are taken as the training set. We train ten models using the procedure outlined above for $3 \times 10^3$ epochs followed by a refinement period of $10^3$ epochs. Hyperparameters used for training are shown in Table S2. Nine of the ten resulting dynamical systems models have two active terms and one has three active terms. The dynamical equations, SINDy coefficient matrix, attractors, and simulated dynamics for two example models are shown in Figure S4b,c. The models with two active terms all have one of the two forms shown in the figure: three models have a linear oscillation and six models have a nonlinear oscillation. Both model forms have similar levels of error on the test set and are able to predict the dynamics in the test set from simulation, as shown in the fourth panel of Figure S4b,c.

**Nonlinear pendulum.** The nonlinear pendulum equation is given by

$$\ddot{z} = -\sin z. \qquad [4]$$

We generate synthetic video of the pendulum in two spatial dimensions by creating high-dimensional snapshots given by

$$x(y_1, y_2, t) = \exp\left(-20\left((y_1 - \cos(z(t) - \pi/2))^2 + (y_2 - \sin(z(t) - \pi/2))^2\right)\right)$$

at a discretization of $y_1, y_2 \in [-1.5, 1.5]$. We use 51 grid points in each dimension resulting in snapshots $\mathbf{x}(t) \in \mathbb{R}^{2601}$. To generate a training set, we simulate Eq. (4) from 100 randomly chosen initial conditions with $z(0) \in [-\pi, \pi]$ and $\dot{z}(0) \in [-2.1, 2.1]$. The initial conditions are selected from a uniform distribution in the specified range but are restricted to conditions for which the pendulum does not have enough energy to do a full loop. This condition is determined by checking that $|\dot{z}(0)^2/2 - \cos z(0)| \leq 0.99$.

Following the training procedure outlined above, we train ten models for $5 \times 10^3$ epochs followed by a refinement period of $10^3$ epochs. Hyperparameters used for this example are shown in Table S3. Five of the ten resulting models correctly recover the nonlinear pendulum equation. These five models have the best performance of the ten models. The attractor and simulated dynamics for the best of these five models are shown in Figure S5. One model, also shown in Figure S5, recovers a linear oscillator. This model is able to achieve a reasonably low prediction error for $\ddot{\mathbf{x}}, \ddot{\mathbf{z}}$ but the simulated dynamics, while still oscillatory, appear qualitatively different from the true pendulum dynamics. The four remaining models all have two active terms in the dynamics and have a worse performance than the models with one active term.

## Interpretability and generalizability of the resulting models

While neural network approaches are a powerful tool for model discovery, they come with the major limitation that the resulting models typically have limited generalizability and interpretability. In this work we have attempted to leverage the power of neural networks to discover classically interpretable models by combining with SINDy. To further clarify this point, here we provide a discussion on the strengths and limitations in this regard.

Our approach results in models with two components: an autoencoder network that represents a coordinate transformation from measurement data to intrinsic coordinates, and a SINDy model that captures the dynamics in the intrinsic space. The coordinate transformation is a neural network model, and it will typically not generalize to data outside of the distribution of the training set. In addition, this coordinate transformation does not improve upon standard neural network approaches with regards to interpretability. While an attempt could be made to interpret the network layers in order to understand more about the coordinate transformation, this would merely be comparable to other attempts at interpreting neural network models, and could borrow from other approaches that aim to interpret NNs that learn coordinate transformations.

The potential for interpretability and generalizability of the approach presented in this paper results from the use of a SINDy model for the intrinsic dynamics. Specifically, the dynamical models are encouraged to be parsimonious with only a few active terms, making them easier to interpret. Parsimonious models also tend to generalize better, as they are less likely

to overfit the training data. SINDy models take on a standard dynamical system form: the model is written as a system of differential equations whose terms are a subset of the chosen library functions. In the examples presented here, the SINDy library contains standard functions including polynomials and trigonometric functions, which are quite general and may have physical interpretations. Beyond providing interpretability, using standard functions in the SINDy model also allows the dynamics to generalize off of the attractor captured by training data. This generalizability may be limited by numerical inaccuracies in estimating the model coefficients and by the fact that the coordinate transformation does not generalize as well, making it difficult to map the intrinsic coordinates back up to the input space. However, the general form of the model can still be used to understand dynamics off of the attractor. To further explore the limits on generalizability, we have applied our Lorenz model to data from several initial conditions outside of the distribution used to generate training data. These results are discussed in the supplemental section on the Lorenz example. In addition to generalizing off attractor, SINDy models may also be used to study new parameter regimes in the dynamics. By separating the coefficient mask, which selects which terms are active in the SINDy model, from the coefficient values, these values can be adjusted to study different behaviors of the same system.

**Partial retraining of the model.** Using a SINDy model for the dynamics provides an additional benefit that increases the generalizability of our approach. This benefit comes from the idea that if the form of the dynamics is known, the retraining problem is greatly simplified. Specifically, we consider the case where a SINDy autoencoder model has been learned for a given system and either of the following changes occurs: the parameters of the underlying dynamics change, or the coordinate transformation changes. While the general form of the underlying dynamics remains the same, the learned SINDy autoencoder model will no longer provide a good model for the data and will likely have poor prediction errors. However, in many instances a new model may quickly be learned by retraining the network while initializing from the previously learned model and fixing the form of the dynamics.

We test this concept on a system based on of our Lorenz example. We combine the two cases discussed above by making two changes to the data generation procedure: (1) we change one of the parameters in the Lorenz equations from $\rho = 28$ to $\rho = 32$, and (2) we change the spatial modes $\mathbf{u}_i$ from Legendre polynomials to Chebyshev polynomials of the first kind. Predictably, a model trained on the original Lorenz dataset results in large errors: the relative $L_2$ errors are $e_{\mathbf{x}} \approx 0.13$, $e_{\dot{\mathbf{x}}} \approx 3.5$, and $e_{\dot{\mathbf{z}}} \approx 4.2$, respectively. However, by initializing with this model and fixing the coefficient mask that defines the form of the dynamics, we can train the network to learn the new mapping in only a few hundred epochs. After 500 epochs of training, the relative $L_2$ errors are $e_{\mathbf{x}} \approx 1.1 \times 10^{-4}$, $e_{\dot{\mathbf{x}}} \approx 3.5 \times 10^{-3}$, and $e_{\dot{\mathbf{z}}} \approx 7.9 \times 10^{-3}$, respectively. This training duration is significantly shorter than the original training period. For initial conditions outside of the training distribution, the retraining decreases the relative $L_2$ errors from $e_{\mathbf{x}} \approx 0.30$, $e_{\dot{\mathbf{x}}} \approx 1.4$, $e_{\dot{\mathbf{z}}} \approx 2.2$ to $e_{\mathbf{x}} \approx 0.017$, $e_{\dot{\mathbf{x}}} \approx 0.17$, and $e_{\dot{\mathbf{z}}} \approx 0.23$, respectively.

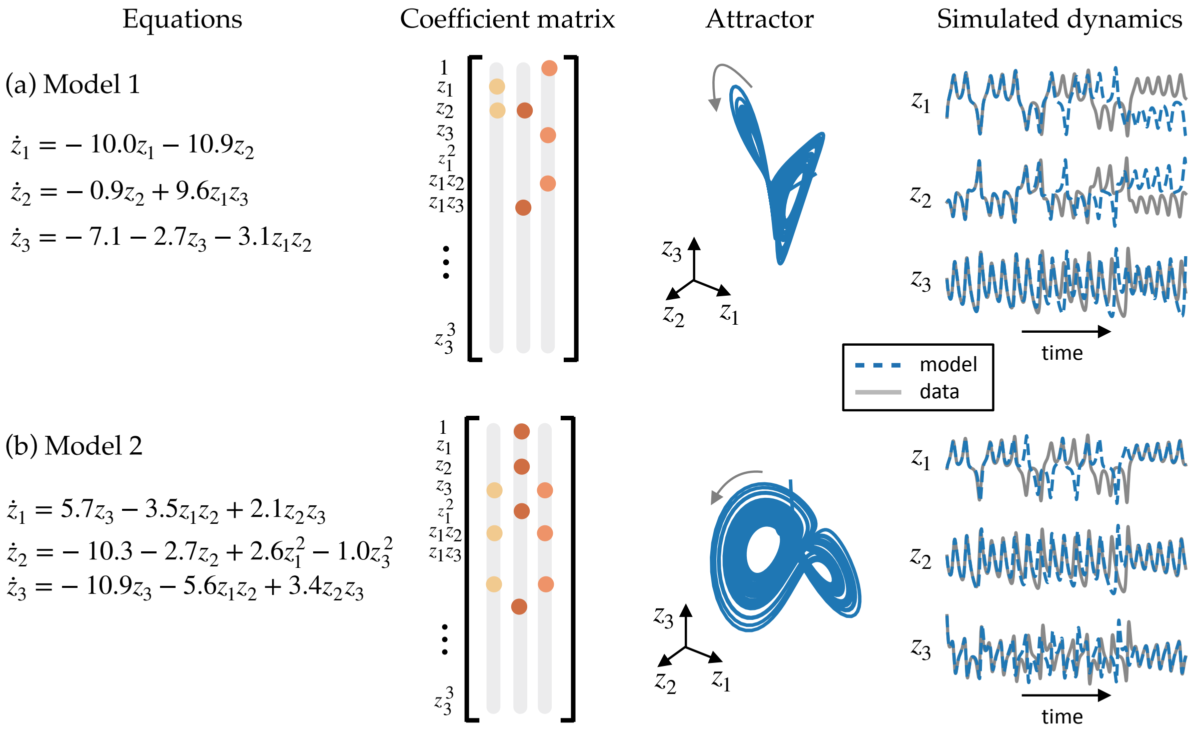


**Fig. S1.** Comparison of two discovered models for the Lorenz example system. For both models we show the equations, SINDy coefficients $\Xi$, attractors, and simulated dynamics for two models discovered by the SINDy autoencoder. (a) A model with 7 active terms. This model can be rewritten in the same form as the original Lorenz system using the variable transformation described in Eq. (2). Simulation of the model produces an attractor with a two lobe structure and is able to reproduce the true trajectories of the dynamics for some time before eventually diverging due to the chaotic nature of the system. (b) A model with 10 active terms. The model has more terms than the true Lorenz system, but has a slightly lower errors $e_{\mathbf{x}}, e_{\dot{\mathbf{x}}}$ than the model in (a). Simulation shows that the dynamics also lie on an attractor with two lobes. The model can accurately predict the true dynamics over a similar duration as (a).

**Table S1. Hyperparameter values for the Lorenz example**

| Parameter | Value |
|---|---|
| n | 128 |
| d | 3 |
| training samples | $5.12 \times 10^5$ |
| batch size | 8000 |
| activation function | sigmoid |
| encoder layer widths | $64, 32$ |
| decoder layer widths | $32, 64$ |
| learning rate | $10^{-3}$ |
| SINDy model order | 1 |
| SINDy library polynomial order | 3 |
| SINDy library includes sine | no |
| SINDy loss weight $\dot{\mathbf{x}}$, $\lambda_1$ | $10^{-4}$ |
| SINDy loss weight $\dot{\mathbf{z}}$, $\lambda_2$ | $0$ |
| SINDy regularization loss weight, $\lambda_3$ | $10^{-5}$ |

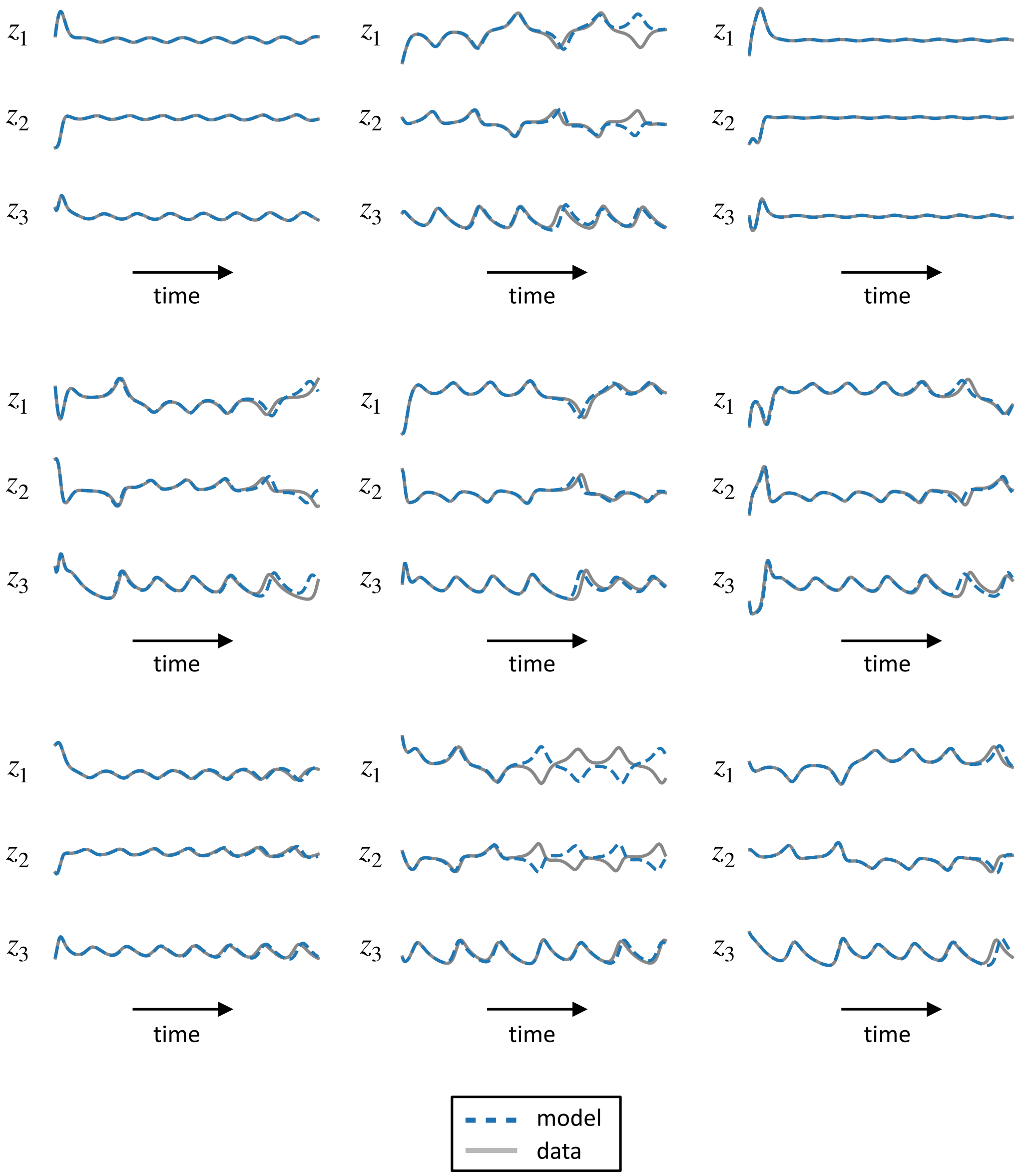


**Fig. S2.** Simulations of the discovered Lorenz model for nine initial conditions sampled from the same distribution as the training set. The simulated trajectories (plotted in blue) are compared with the low-dimensional trajectories found by passing the high-dimensional input data through the encoder (plotted in gray). Simulations are from $t = 0$ to $5$, the same duration used for each trajectory in the training set.

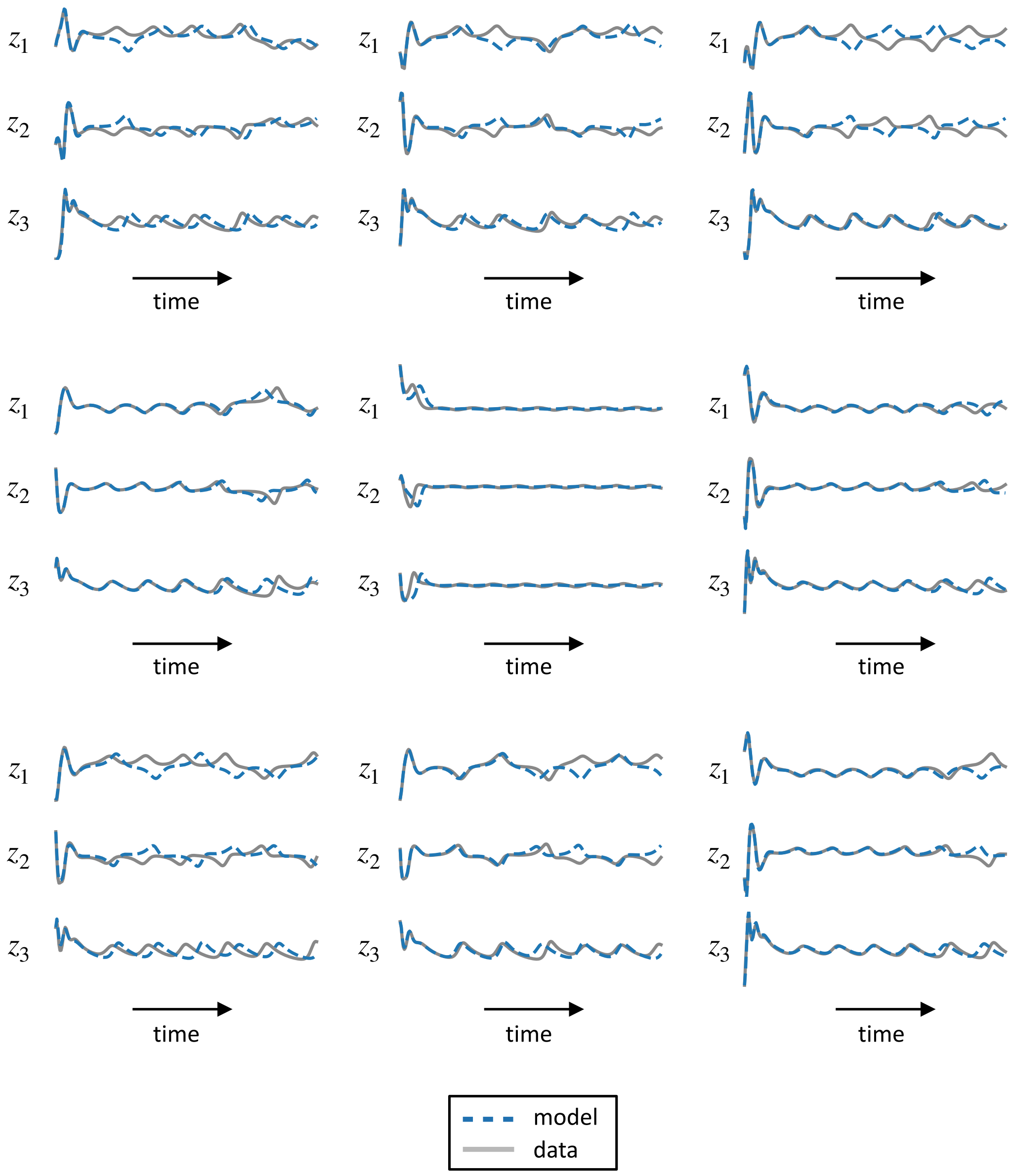


**Fig. S3.** Simulations of the discovered Lorenz model for nine initial conditions sampled from outside of the distribution used in the training set. The initial conditions are sampled from a region surrounding the training distribution. As in Figure S2, the simulated trajectories (blue) are compared with the low-dimensional trajectories found via the encoder (plotted in gray). Simulations are from $t = 0$ to 5.

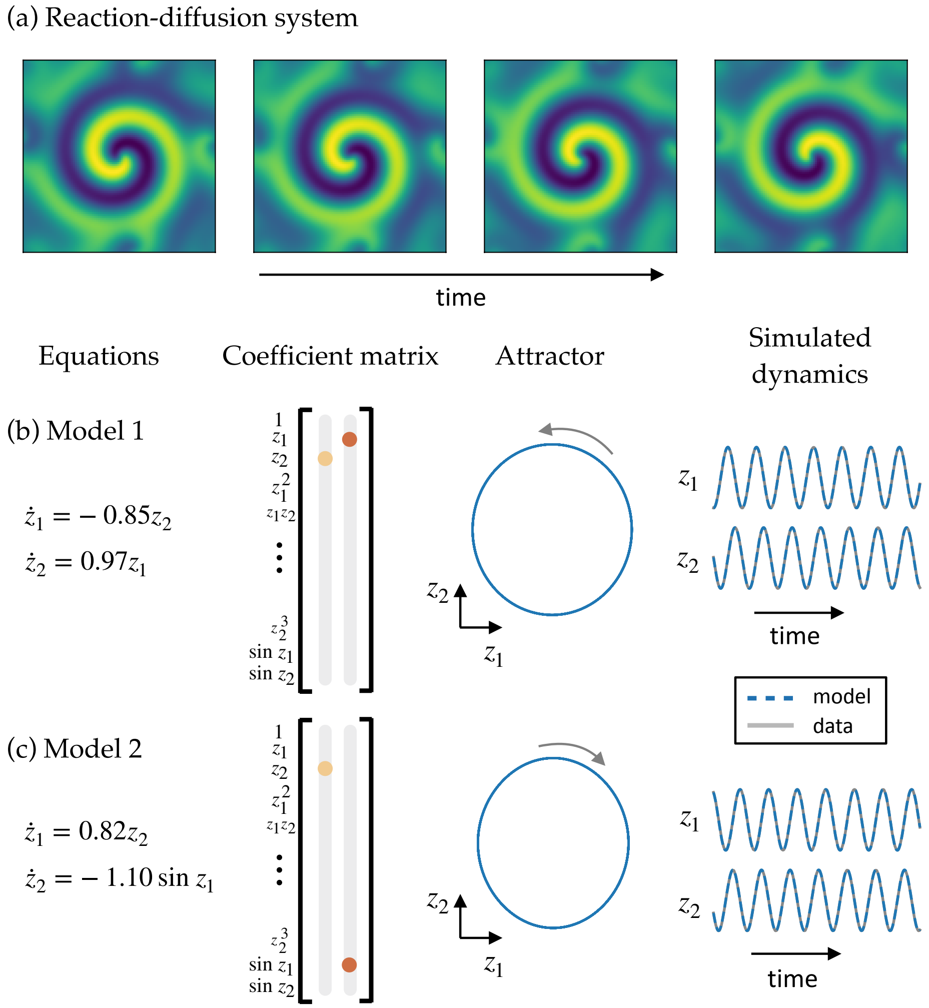


**Fig. S4.** Resulting models for the reaction-diffusion system. (a) Snapshots of the high-dimensional system show a spiral wave formation. (b,c) Equations, SINDy coefficients $\Xi$, attractors, and simulated dynamics for two models discovered by the SINDy autoencoder. The model in (b) is a linear oscillation, whereas the model in (c) is a nonlinear oscillation. Both models achieve similar error levels and can predict the dynamics in the test set via simulation of the low-dimensional dynamical system.

**Table S2. Hyperparameter values for the reaction-diffusion example**

| **Parameter** | **Value** |
|---|---|
| n | $10^4$ |
| d | 2 |
| training samples | 8000 |
| batch size | 1024 |
| activation function | sigmoid |
| encoder layer widths | $256$ |
| decoder layer widths | $256$ |
| learning rate | $10^{-3}$ |
| SINDy model order | 1 |
| SINDy library polynomial order | 3 |
| SINDy library includes sine | yes |
| SINDy loss weight $\dot{\mathbf{x}}$, $\lambda_1$ | $0.5$ |
| SINDy loss weight $\dot{\mathbf{z}}$, $\lambda_2$ | $0.01$ |
| SINDy regularization loss weight, $\lambda_3$ | $0.1$ |

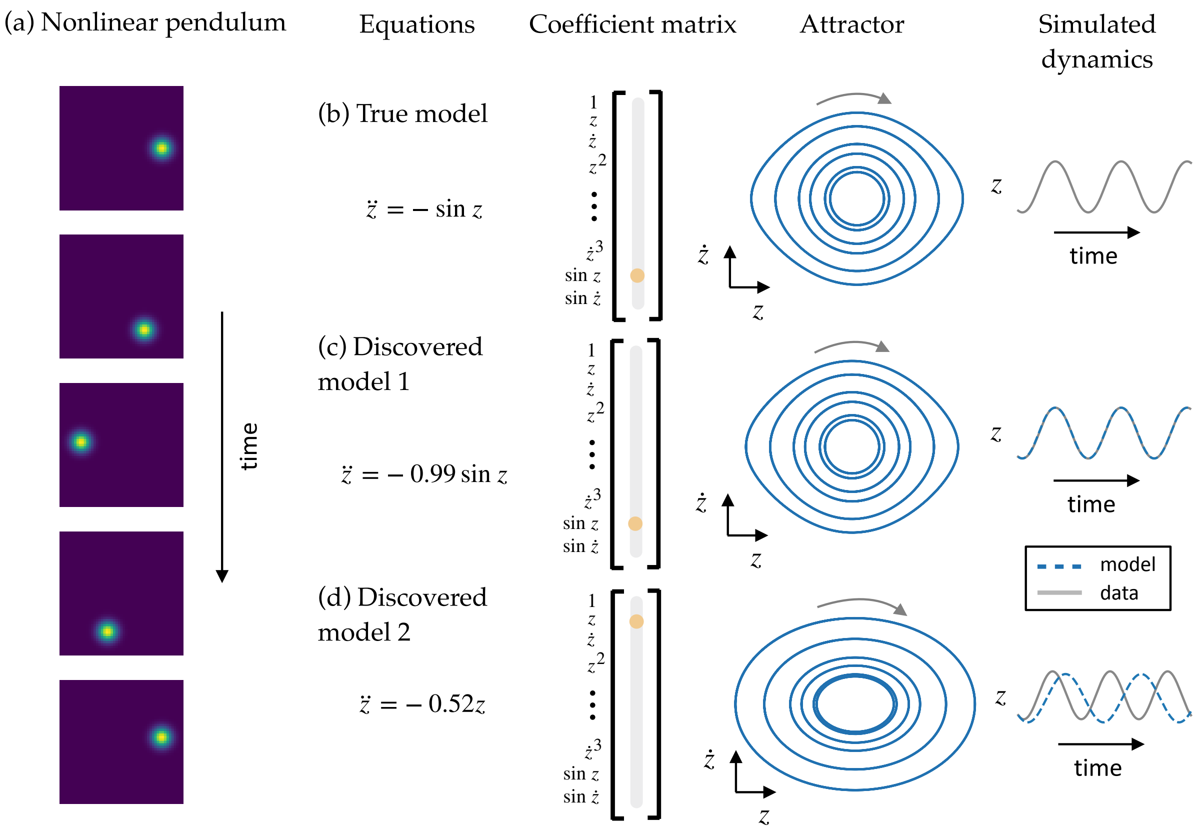


**Fig. S5.** Resulting models for the nonlinear pendulum. (a) Snapshots of the high-dimensional system are images representing the position of the pendulum in time. (b,c,d) Comparison of two discovered models with the true pendulum dynamics. Equations, SINDy coefficients $\Xi$, attractors, and simulated dynamics for the true pendulum equation are shown in (b). The model in (c) correctly discovered the true form of the pendulum dynamics. Both the image of the attractor and simulations match the true dynamics. (d) In one instance of training, the SINDy autoencoder discovered a linear oscillation for the dynamics. This model achieves a worse error than the model in (c).

**Table S3. Hyperparameter values for the nonlinear pendulum example**

| **Parameter** | **Value** |
|---|---|
| n | $2601$ |
| d | 1 |
| training samples | $5 \times 10^4$ |
| batch size | 1024 |
| activation function | sigmoid |
| encoder layer widths | $128, 64, 32$ |
| decoder layer widths | $32, 64, 128$ |
| learning rate | $10^{-4}$ |
| SINDy model order | 2 |
| SINDy library polynomial order | 3 |
| SINDy library includes sine | yes |
| SINDy loss weight $\dot{x}$, $\lambda_1$ | $5 \times 10^{-4}$ |
| SINDy loss weight $\dot{z}$, $\lambda_2$ | $5 \times 10^{-5}$ |
| SINDy regularization loss weight, $\lambda_3$ | $10^{-5}$ |

## References


1. Glorot X, Bengio Y (2010) Understanding the difficulty of training deep feedforward neural networks in *Proceedings of the thirteenth international conference on artificial intelligence and statistics*. pp. 249–256.
2. Kingma DP, Ba J (2014) Adam: A method for stochastic optimization. *CoRR* abs/1412.6980.
3. Tibshirani R, Wainwright M, Hastie T (2015) *Statistical learning with sparsity: the lasso and generalizations*. (Chapman and Hall/CRC).
4. Van Nguyen H, Patel VM, Nasrabadi NM, Chellappa R (2012) Kernel dictionary learning in *IEEE International Conference on Acoustics, Speech and Signal Processing*. pp. 2021–2024.
5. Abadi M, et al. (2016) Tensorflow: A system for large-scale machine learning in *12th USENIX Symposium on Operating Systems Design and Implementation (OSDI 16)*. pp. 265–283.
